# Supplementary material for: Adipose and Plasma microRNAs miR-221 and 222 Associate with Obesity, Insulin Resistance, and New Onset Diabetes after Peritoneal Dialysis
Source: Nutrients. 2022 Nov 18;14(22):4889. doi: 10.3390/nu14224889 (PMC9699429; doi:10.3390/nu14224889)
Supplement: Supplementary file 1 [file nutrients-14-04889-s001.zip › nutrients-1978763-supplementary.pdf]

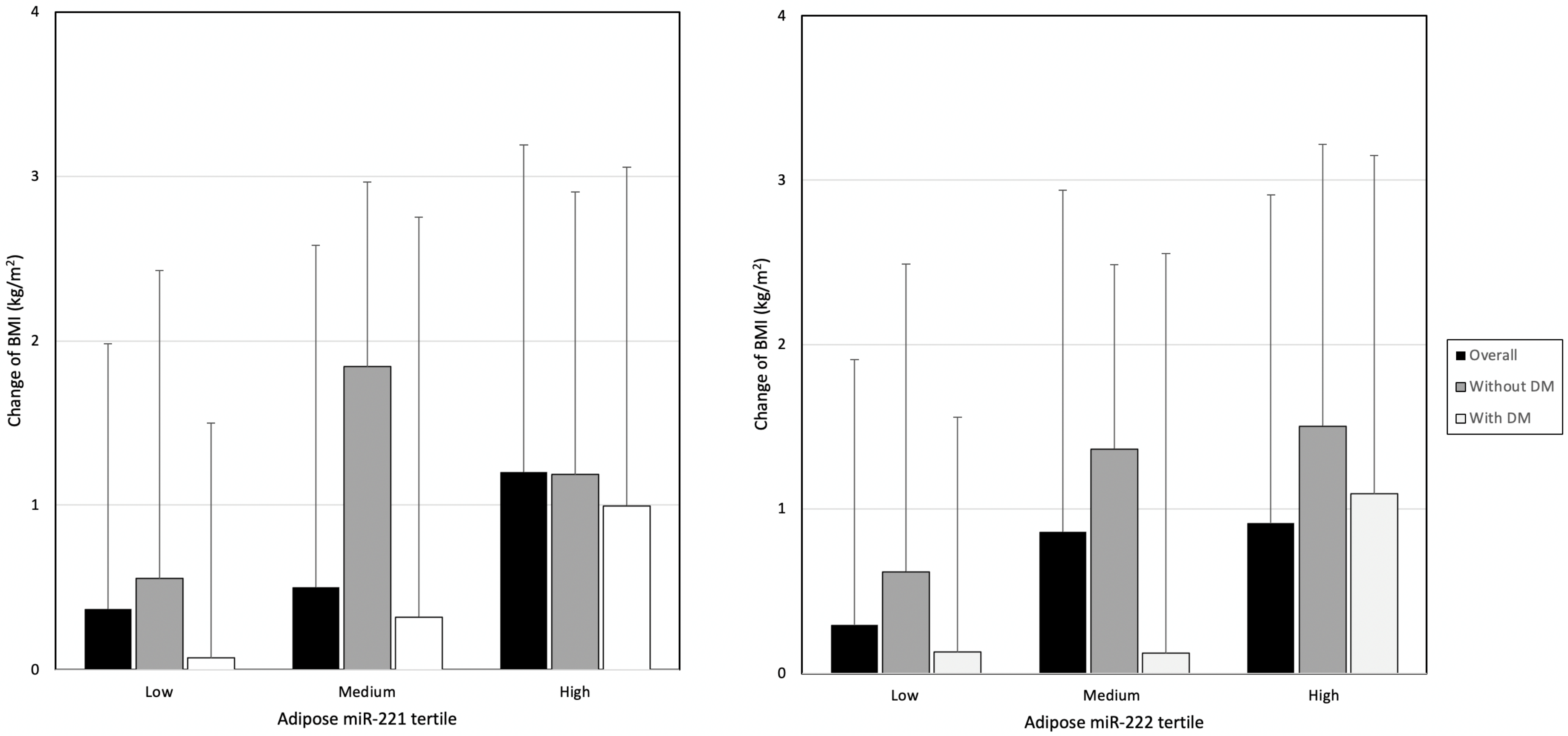

Supplementary Figure S1. BMI change according to adipose miR-221 & 222 tertiles.

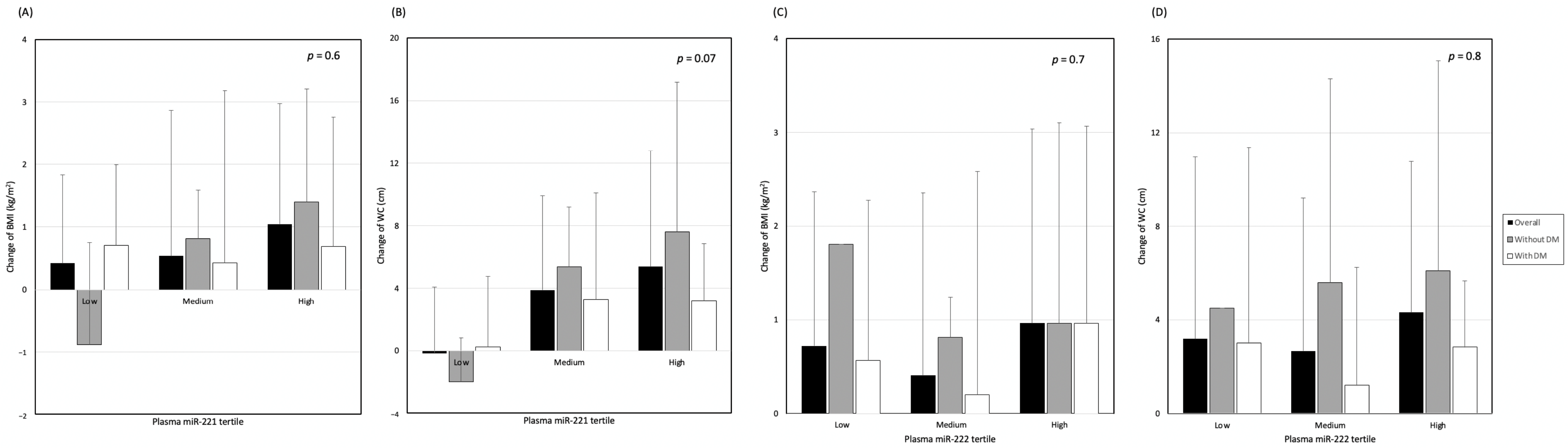

Supplementary Figure S2. BMI and WC changes according to plasma (A & B) miR-221 & (C & D) 222 tertiles.

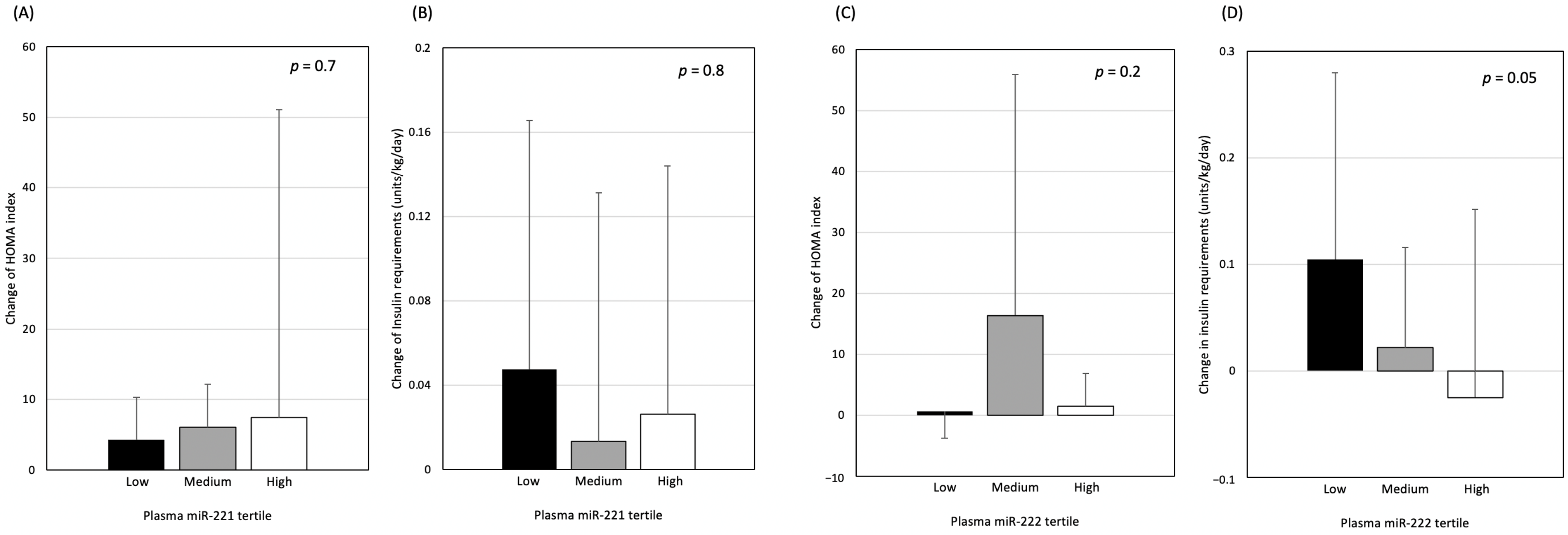

Supplementary Figure S3. HOMA and daily insulin requirements changes according to plasma (A & B) miR-221 & (C & D) 222 tertiles.

Supplementary Table S1. Correlations between miR-221 & 222 with plasma glycemic profile and nutrition.

	miR-221		miR-222	
	Adipose	Plasma	Adipose	Plasma
Fasting glucose	r = -0.03<br>*p* = NS	r = -0.32<br>*p* = 0.02	r = 0.01<br>*p* = NS	r = -0.24<br>*p* = NS
HbA1c	r = 0.23<br>*p* = 0.005	r = -0.33<br>*p* = 0.02	r = 0.28<br>*p* = 0.001	r = -0.31<br>*p* = 0.02
Fasting insulin	r = 0.15<br>*p* = NS	r = -0.04<br>*p* = NS	r = 0.20<br>*p* = 0.015	r = -0.25<br>*p* = NS
HOMA	r = 0.15<br>*p* = NS	r = -0.14<br>*p* = NS	r = 0.21<br>*p* = 0.01	r = -0.03<br>*p* = 0.015
Albumin	r = 0.03<br>*p* = NS	r = 0.36<br>*p* = 0.006	r = 0.06<br>*p* = NS	r = 0.30<br>*p* = 0.03
BMI	r = 0.06<br>*p* = NS	r = 0.03<br>*p* = NS	r = 0.1<br>*p* = NS	r = 0.005<br>*p* = NS
WC	r = 0.05<br>*p* = NS	r = -0.06<br>*p* = NS	r = 0.08<br>*p* = NS	r = 0.04<br>*p* = NS

Data were compared by Spearman's rank correlation coefficient.

HbA1c, glycated haemoglobin A1c; HOMA, homeostatic model assessment; BMI, body mass index; WC, waist circumference; NS, not significant.

Supplementary Table S2. Clinical characteristics of patients developed new onset diabetes mellitus (NODM) after peritoneal dialysis.

	With NODM (n = 26)	Without NODM (n = 35)	P-value
Age	52.4 ± 15.4	55.3 ± 10.8	$p = 0.4$[a]
Male sex, No. (%)	19 (73.1%)	21 (60.0%)	$p = 0.3$[b]
Primary renal disease			$p = 0.3$[b]
Diabetes mellitus	7 (26.9%)	5 (14.3%)	
Hypertension	11 (42.3%)	16 (45.7%)	
Glomerulonephritis	0 (0%)	1 (2.9%)	
Polycystic kidney disease	4 (15.4%)	2 (5.7%)	
Urological	0 (0%)	2 (5.7%)	
Others	4 (15.4%)	9 (25.7%)	
Comorbidities			
Ischemic heart disease	3 (11.5%)	4 (11.4%)	$p = 0.9$[b]
Cerebrovascular accident	5 (19.2%)	4 (11.4%)	$p = 0.4$[b]
Charlson comorbidities index	4.2 ± 2.3	4.3 ± 1.7	$p = 0.9$[a]
Residual GFR (ml/min/1.73m^2)	3.4 ± 2.4	2.9 ± 2.2	$p = 0.5$[a]
Laboratory parameters			
Urea (mmol/L)	28.1 ± 8.7	33.4 ± 6.7	$p = 0.01$[a]
Creatinine (umol/L)	908 ± 281	931 ± 325	$p = 0.8$[a]
Albumin (g/L)	34.6 ± 5.3	37.1 ± 3.8	$p = 0.048$[a]
High-sensitive C-reactive protein (mg/L)	22.2 ± 35.7	9.9 ± 30.9	$p = 0.2$[a]
Peritoneal characteristics			

D/P4	0.69 ± 0.12	0.69 ± 0.11	p = 0.9[a]
MTAC	11.0 ± 5.6	11.6 ± 5.0	p = 0.7[a]
Anthropometry			
Body weight (kg)	66.3 ± 18.5	58.4 ± 10.2	p = 0.05[a]
Body height (m)	163 ± 11	162 ± 8	p = 0.7[a]
BMI (kg/m^2)	24.7 ± 5.4	22.2 ± 3.1	p = 0.04[a]
WC (cm)	88.7 ± 13.5	79.9 ± 10.2	p = 0.009[a]
Glycemic profile			
HbA1c (%)	5.5 ± 0.5	5.4 ± 0.4	p = 0.6[a]
Fasting glucose (mmol/L)	5.0 ± 0.8	4.8 ± 0.4	p = 0.2[a]
Fasting insulin (mIU/L)	16.1 ± 14.1	11.2 ± 5.8	p = 0.09[a]
C-peptide (ng/mL)	11.6 ± 9.4	9.1 ± 8.3	p = 0.3[a]
HOMA	3.5 ± 2.8	2.4 ± 1.4	p = 0.07[a]

Data are presented as mean ± standard deviation and compared by paired Student's t test[a] and chi-square test[b].

NODM, new onset diabetes mellitus; GFR, glomerular filtration rate, D/P4, dialysate-to-plasma ratio of creatinine at 4 hours; MTAC, mass transfer area coefficient of creatinine; BMI, body mass index; WC, waist circumference; HbA1c, glycated haemoglobin A1c; HOMA, homeostatic model assessment.
